# Thiol-Associated Antioxidant Activity of Recombinant Mussel Foot Protein Mfp6-1 Supports Cutaneous Wound Repair in a Murine Model

**DOI:** 10.3390/md24050157

**Published:** 2026-04-29

**Authors:** Zi-Jun Li, Kun-Cheng Wang, Zhi-Ming Shen, Yu-Qing Wang, Yi-Feng Li

**Affiliations:** 1 Key Laboratory of Exploration and Utilization of Aquatic Genetic Resources, Ministry of Education, Shanghai Ocean University, Shanghai 201306, China; m230100118@st.shou.edu.cn (Z.-J.L.); m240100407@st.shou.edu.cn (Z.-M.S.); d220100011@st.shou.edu.cn (Y.-Q.W.); 2 International Research Centre for Marine Biosciences, Ministry of Science and Technology, Shanghai Ocean University, Shanghai 201306, China; 3 Shanghai Collaborative Innovation Center for Cultivating Elite Breeds and Green-Culture of Aquaculture Animals, Shanghai 201306, China; m240250801@st.shou.edu.cn; 4 College of Marine Living Resource Sciences and Management, Shanghai Ocean University, Shanghai 201306, China

**Keywords:** mussel foot protein 6, antioxidant, thiol, wound healing, tissue regeneration

## Abstract

Mussel foot proteins (Mfps) are renowned for their underwater adhesion, whereas their biotechnological potential for cutaneous wound repair remains largely underexplored. In this study, we identified and characterized a cysteine-rich mussel foot protein, Mfp6-1, from *Mytilus coruscus* and investigated its therapeutic potential for wound healing. Sequence analysis showed that Mfp6-1 is enriched in cysteine (11.0%) and tyrosine (~16.5%). We successfully expressed recombinant Mfp6-1 (rMfp6-1) in *E. coli*. Structural prediction based on the mature peptide sequence suggested that rMfp6-1 adopts a relatively compact fold containing several short β-structural elements. In vitro assays demonstrated that rMfp6-1 possesses antioxidant activity in the 2,2-diphenyl-1-picrylhydrazyl (DPPH) assay, and alkylation experiments suggested that cysteine residues contribute importantly to this activity. Dithio-bis-nitrobenzoic acid (DTNB)-based thiol quantification further demonstrated that rMfp6-1 contained abundant accessible free sulfhydryl groups, supporting an important contribution of cysteine-derived thiols to its antioxidant activity. Experiments on a full-thickness mouse wound model showed that rMfp6-1 treatment resulted in significantly faster wound contraction. Morphological analysis further revealed that rMfp6-1 optimizes the healing microenvironment by promoting collagen accumulation and re-epithelialization. Additionally, the treatment was found to trigger vascular endothelial growth factor (VEGF)-mediated angiogenesis, thereby improving the overall quality of the regenerated tissue. Furthermore, rMfp6-1 treatment significantly reduced interleukin-6 (IL-6) expression, suggesting that its antioxidant capacity creates a permissive microenvironment for tissue regeneration by suppressing excessive inflammation. These findings indicate that recombinant rMfp6-1 is a promising bioactive candidate for wound-healing applications.

## 1. Introduction

Mussels are able to remain firmly attached in the intertidal zone despite constant wave action, a capability that originates from their byssus system [1]. The mussel foot secretes multiple mussel foot proteins (Mfps) that assemble into the byssal thread, comprising a collagenous core and a terminal adhesive plaque, thereby achieving strong wet adhesion to various substrates [2,3]. The Mfp family exhibits clear functional diversification: Mfp3 and Mfp5 are located at the plaque–substrate interface and mediate adhesion; Mfp2 and Mfp4 contribute to structural organization and cross-linking; and Mfp1 forms the outer coating, enhancing wear resistance and fatigue durability [4,5,6]. Among these, Mfp6 is notably rich in cysteine residues and shows exceptional antioxidant activity [7]. Within the acidic, high-ionic-strength microenvironment of the plaque, Mfp6 reduces oxidized 3,4-dihydroxy-L-phenylalanine (DOPA)-quinone back to DOPA and scavenges reactive oxygen species (ROS), thereby preserving the chemical stability and adhesive strength of the byssal plaque [8,9].

The redox-buffering strategy of Mfp6 offers valuable inspiration for biomedical contexts in which oxidative stress compromises tissue integrity and repair. Upon wounding, platelet aggregation and clot formation temporarily seal the injury and initiate the inflammatory phase [10]. Neutrophils arrive first, followed by monocytes that differentiate into macrophages, and these immune cells collectively secrete proteolytic enzymes, pro-inflammatory cytokines, and large amounts of reactive oxygen species (ROS) to eliminate invading pathogens [11]. However, excessive ROS can damage extracellular matrix components, perturb gene expression, and delay wound closure [12]. Under physiological conditions, antioxidant enzymes such as superoxide dismutase, catalase, and glutathione peroxidase maintain ROS homeostasis [13], yet this defense is often insufficient under pathological conditions such as diabetes or infection, making exogenous antioxidants desirable. Unlike enzymatic antioxidants whose activity is easily affected by pH or oxidative environments [14], Mfp6 can preserve DOPA residues in a reduced state even under saline, wet conditions [8,9]. These properties make Mfp6 an attractive molecular model for designing bioinspired materials that integrate durable wet adhesion with redox-regulating capability—an approach particularly suited to promoting tissue repair in oxidative wound microenvironments.

Mfp6-1 was selected for the present study because mussel Mfp6 family members have been associated with redox regulation in the byssal plaque [7,8], and the cysteine- and tyrosine-rich sequence features of Mfp6-1 suggest its potential relevance to antioxidant-related bioactivity. Building on this rationale, we characterized the gene sequence, amino acid composition, and secondary/tertiary structures of Mfp6-1 from the mussel *Mytilus coruscus*. We achieved recombinant expression of Mfp6-1 in Escherichia coli via codon optimization to validate its antioxidant function. Recombinant Mfp6-1 (rMfp6-1) exhibited significant antioxidant activity in DPPH assays, outperforming our previously reported mussel foot protein Mfp20 [15]. DTNB-based thiol quantification further showed that rMfp6-1 contains accessible free sulfhydryl groups, supporting the view that rMfp6-1 contributes to its antioxidant-related activity. In a full-thickness dorsal skin defect model in mice, comprehensive evaluation of wound closure kinetics, HE/Masson histology, and immunohistochemical markers of inflammation, including IL-6 and tumor necrosis factor-alpha (TNF-α), and angiogenesis (VEGF) showed that rMfp6-1 attenuated early inflammation, accelerated wound repair, and improved tissue quality post-healing. Collectively, rMfp6-1 functions as a promising antioxidant candidate for efficient skin wound repair.

## 2. Results

### 2.1. Features of Mfp6-1 Sequence

**Figure 1:**
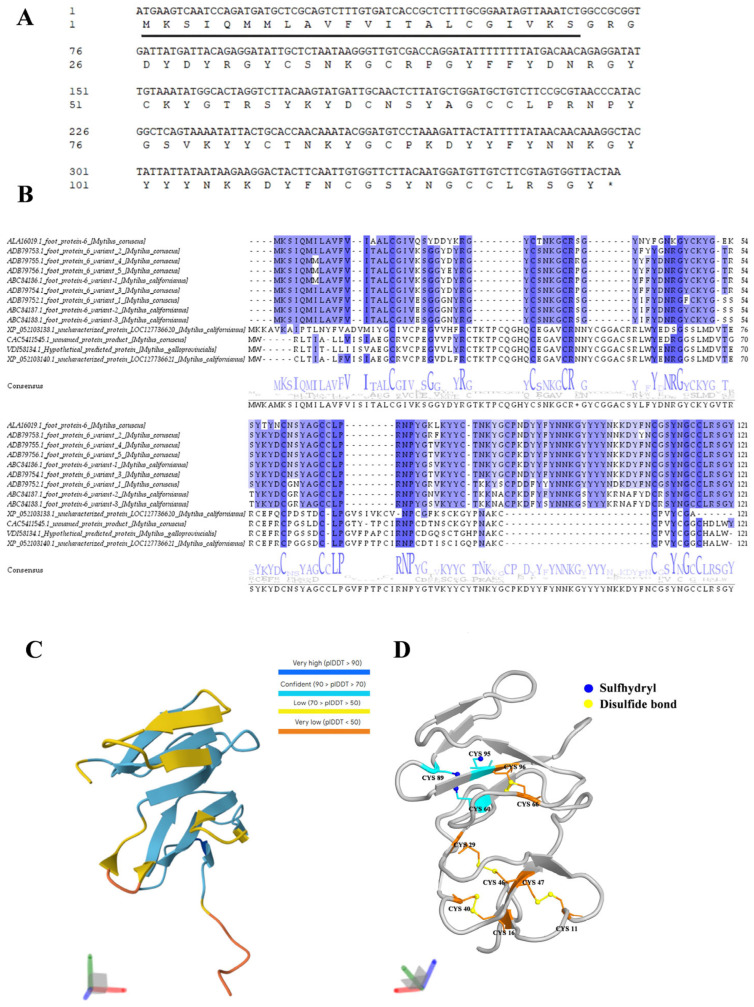
Sequence and structural characterization of Mfp6-1. (**A**) Nucleotide and deduced amino acid of natural *M. coruscus* Mfp6-1. The signal peptide is underlined, and the stop codon is indicated by an asterisk (*). (**B**) Multiple sequence alignment of Mfp6 homologs from representative *Mytilus* species. Conserved residues were highlighted by color coding, and consensus sequence is shown below the alignment. (**C**) AlphaFold-predicted three-dimensional structure of mature Mfp6-1 colored by pLDDT confidence scores, with dark blue indicating very high confidence (pLDDT > 90), light blue indicating confident regions (70 < pLDDT ≤ 90), yellow indicating low confidence (50 < pLDDT ≤ 70), and orange-red indicating very low confidence (pLDDT ≤ 50). (**D**) Structural distribution of cysteine residues in the predicted model. Putative free sulfhydryl sites are shown in blue/cyan, whereas putative disulfide-bonded cysteines are shown in yellow/orange.

The *M. coruscus* Mfp6-1 gene contains a 372-bp full-length cDNA with an open reading frame encoding a 123-residue precursor peptide. Sequence analysis showed that the precursor comprises a 22-residue signal peptide and a 101-residue mature peptide (Figure 1A). The predicted mature peptide has a predicted molecular weight of 12.04 kDa and a theoretical isoelectric point (pI) of 9.04.

Blastp and multiple sequence alignment showed that Mfp6-1 shares high sequence similarity with homologous proteins from several *Mytilus* species (Figure 1B). The signal peptide region was generally conserved, and the mature peptide displayed a characteristic Cys/Tyr-rich pattern (Figure 1B). SMART analysis identified an N-terminal signal peptide (residues 1–22) and a low-complexity region but did not detect any well-defined canonical conserved domain.

To characterize the structural features of Mfp6-1, the mature peptide sequence was analyzed using AlphaFold. The predicted model suggested a relatively compact cysteine-rich fold containing several short β-structural elements, whereas the terminal and loop regions appeared more flexible (Figure 1C). Overall, the model showed moderate confidence: most residues were assigned to the confident or low-confidence ranges, including 59 residues with 70 < pLDDT ≤ 90 and 35 residues with 50 < pLDDT ≤ 70, whereas only 2 and 6 residues fell into the very high- and very low-confidence categories, respectively.

Based on the AlphaFold predicted model [16], Cys11, Cys16, Cys29, Cys40, Cys46, Cys47, Cys66, and Cys96 were putative disulfide-bonded cysteine residues, whereas Cys60, Cys89, and Cys95 were potentially exposed cysteine residues (Figure 1D).

### 2.2. Expression, Purification, and Antioxidant Properties of Recombinant Mfp6-1 (rMfp6-1)

**Figure 2:**
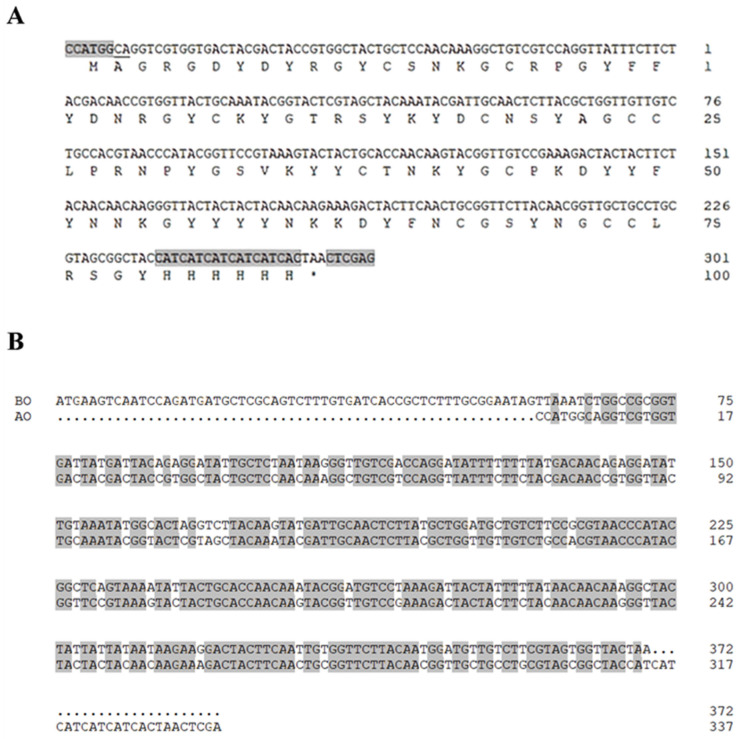
Sequence architecture and optimization strategy for rMfp6-1. (**A**) Predicted amino acid sequence of the recombinant Mfp6-1 protein. The recognition sequences for *Nco I* (“CCATGG”) and *Xho I* (“CTCGAG”) are indicated, flanked by protective bases (“CA”). The hexahistidine (6 × His) tag is encoded by “CATCATCATCATCATCAC”. (**B**) Comparative alignment for codon optimization. Sequence homology between the pre-optimized (BO) and post-optimized (AO) genes was evaluated using DNAMAN (v9.0.1.116). Within the alignment, identical nucleotides are indicated by shaded regions, while dots (‘.’) denote gaps in the sequence.

**Figure 3:**
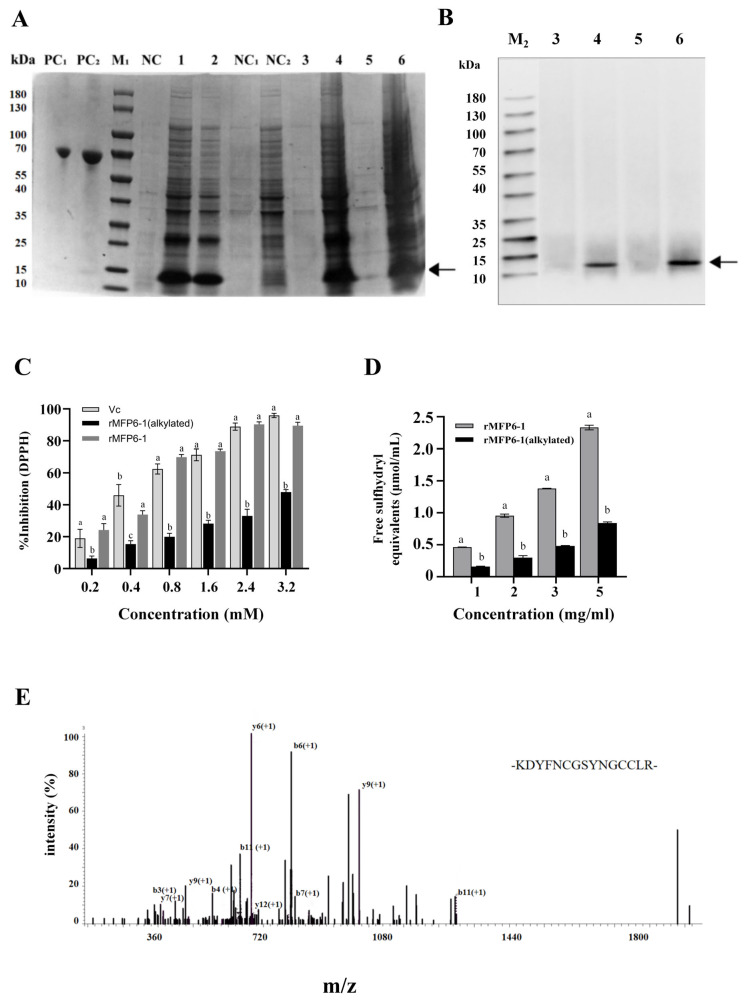
Characterization and functional assay of rMfp6-1. (**A**) SDS-PAGE (Coomassie-stained) and (**B**) Western blot profiling of rMfp6-1. Lanes M1/M2: molecular weight markers; PC1/PC2: BSA standards (1 and 2 μg, respectively); NC: blank cell lysate control. Uninduced samples are represented by NC1 (supernatant) and NC2 (debris). Lanes 1–2 show total cell lysates under different induction conditions (25 °C/10 h and 30 °C/6 h), while the corresponding soluble supernatants (lanes 3, 5) and insoluble pellet fractions (lanes 4, 6) are also displayed. Arrows denote the target bands (~12 kDa). (**C**) Antioxidant capacity of rMfp6-1 and its IAM-alkylated derivative evaluated via DPPH assay, using L-ascorbic acid (Vc) as a reference. (**D**) Quantification of accessible free sulfhydryl equivalents in untreated and IAM-alkylated rMfp6-1 using the DTNB assay. (**E**) MS/MS identification of the tryptic peptide “-KDYFNCGSYNGCCLR-” (*m*/*z* 1765.77541). Data are presented as mean ± SD (*n* = 6). Different letters above the column indicate statistically significant differences (*p* < 0.05).

To facilitate heterologous expression, the mature *Mfp6-1* sequence was codon-optimized for the *E. coli* host (nucleotide and translated sequences illustrated in Figure 2A). This optimization resulted in a 72% identity with the native gene (Figure 2B). The target protein was successfully expressed using the pET-28a vector system. As shown by SDS-PAGE, rMfp6-1 (molecular weight ~12.9 kDa) primarily accumulated in the inclusion bodies (Figure 3A). Based on a comparative analysis with bovine serum albumin (BSA) standards (lanes PC1 and PC2 in Figure 3A), the expression yield of rMfp6-1 was estimated at 80 mg/L of culture. The final recovery after purification and refolding was approximately 23 mg/L of culture. The identity of the 6 × His-tagged fusion protein was further confirmed via Western blotting (Figure 3B).

The radical-scavenging performance of rMfp6-1 was evaluated through the DPPH assay, using L-ascorbic acid as a benchmark. To elucidate the contribution of thiol groups, rMfp6-1 was also tested in its alkylated form (rMfp6-1-IAM). Our findings revealed a dose-dependent scavenging activity of rMfp6-1 against DPPH radicals (Figure 3C). Notably, at 3.2 mM, the inhibitory potency of the rMfp6-1 protein was nearly equivalent to that of L-ascorbic acid. While the alkylated variant also showed a concentration-responsive effect, its scavenging efficiency was significantly reduced compared to unmodified rMfp6-1 (*p* < 0.05; Figure 3C), underscoring the indispensable role of cysteine residues in the protein’s antioxidant mechanism.

To further assess the availability of reactive thiol groups in rMfp6-1, the accessible free sulfhydryl content was quantified using the DTNB assay. The free sulfhydryl equivalents of untreated rMfp6-1 increased progressively with protein concentration, reaching 0.4646 ± 0.0024, 0.9619 ± 0.0277, 1.3831 ± 0.0038, and 2.3481 ± 0.0110 μmol/mL at 1, 2, 3, and 5 mg/mL of rMfp6-1, respectively (Figure 3D). By dividing the free thiol concentration by the protein concentration, the number of accessible free thiol groups per rMfp6-1 molecule was estimated to be approximately 5, based on the theoretical molecular weight of 12.04 kDa [17]. In contrast, IAM treatment markedly reduced the DTNB-detectable sulfhydryl signal at all tested concentrations (Figure 3D).

Following Ni-NTA affinity purification and a gradient urea dialysis refolding process, rMfp6-1 was subjected to MS/MS analysis. A specific tryptic peptide (*m*/*z* 1765.7754 Da) was identified as ‘-KDYFNCGSYNGCCLR-’, matching the 62–76 residue segment of rMfp6-1 (Figure 3E). These data collectively validate the precise synthesis of rMfp6-1 for subsequent functional assays.

### 2.3. Wound Healing Examinations In Vivo

**Figure 4:**
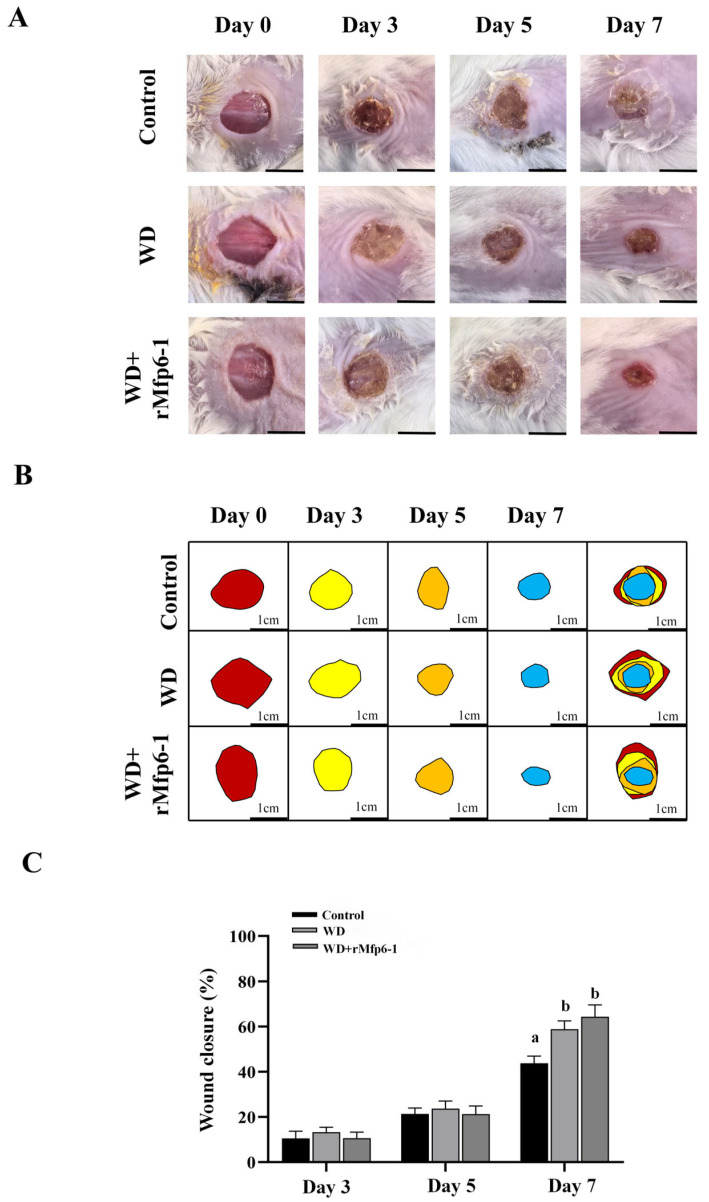
Evaluation of wound healing activity in a murine dorsal skin defect model. (**A**) Representative macroscopic photographs of wounds in the control, WD (wound dressing), and WD + rMfp6-1 (wound dressing + rMfp6-1) groups on days 0, 3, 5, and 7. Scale bar = 1 cm. (**B**) Schematic tracings of wound areas at different time points, visualizing the process of wound contraction (Red: Day 0; Yellow: Day 3; Orange: Day 5; Blue: Day 7). The rightmost column displays an overlay of the wound areas across all time points. (**C**) Quantitative analysis of wound closure percentage on days 3, 5, and 7. Data are presented as mean ± SD (*n* = 6). Different letters above the bars indicate statistically significant differences among groups at the same time point (*p* < 0.05).

To evaluate whether recombinant Mfp6-1 promotes wound healing, a full-thickness excisional skin wound model was established on the dorsal surface of mice, and wound repair was monitored at different time points (Figure 4). Representative images of the wound healing process are shown in Figure 4A. As shown in Figure 4B, semi-quantitative analysis of wound area using ImageJ further confirmed that, relative to the control group, both WD + rMfp6-1 and WD markedly reduced the wound area on day 7. As shown in Figure 4C, on day 7, the wound closure rate in the recombinant Mfp6-1 dressing group (WD + rMfp6-1) and wound dressing (WD) groups was significantly higher than that in the control group (*p* < 0.05). The WD + rMfp6-1 group achieved wound closure rates of 10.66% on day 3, 21.26% on day 5, and 64.41% on day 7, whereas the control group exhibited closure rates of 10.57%, 21.37%, and 43.78% on days 3, 5, and 7, respectively. In addition, the wound dressing (WD) group showed closure rates of 13.29%, 23.72%, and 58.85% on days 3, 5, and 7, respectively.

### 2.4. Histological Evaluation Reveals Enhanced Wound Healing Quality by WD + rMfp6-1

**Figure 5:**
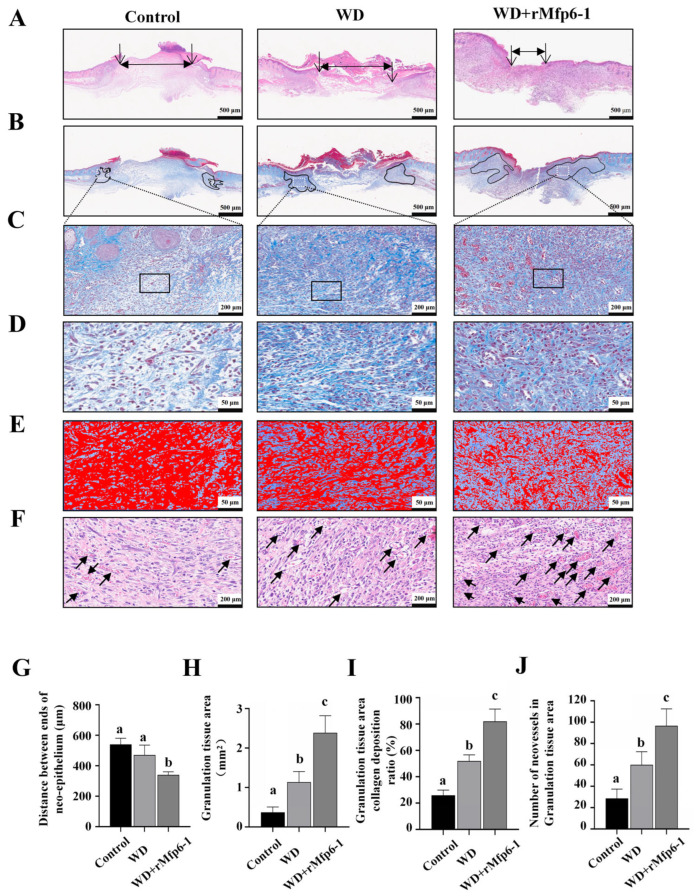
Promotion of re-epithelialization, granulation tissue formation, collagen deposition, and angiogenesis by rMfp6-1. (**A**) Representative images of H&E-stained wound sections showing the distance between the migrating epithelial tongues (indicated by double-headed arrows). (**B**) Masson’s trichrome staining of full-thickness wound sections. Black line outlined areas indicate the granulation tissue. (**C**) Higher magnification images of the white line boxed areas in (**B**) showing granulation tissue. (**D**) Higher magnification images of the black line boxed areas in (**C**). (**E**) False-color images representing collagen content processed for quantitative analysis. Blue represents the collagen area. (**F**) H&E staining showing neovascularization in the granulation tissue. Black arrows indicate newly formed blood vessels. (**G**) Distance between ends of neo-epithelium in (**A**); (**H**) Granulation tissue area calculated from black line area of (**B**); (**I**) Percentage of collagen deposition in granulation tissue area in (**B**) with 48 random fields of view; (**J**) Number of neovessels in the granulation tissue area in (**B**). Different letters above the column indicate statistically significant differences (*p* < 0.05). Results are expressed as mean ± SD (*n* = 6).

To evaluate the microstructural changes in wound healing, we performed H&E and Masson trichrome staining analyses at different time points (Figure 5). As shown in Figure 5A,G, the H&E staining results demonstrated that the WD + rMfp6-1 group had a more significant decrease in the distance among neo-epithelial tongues than the control and WD groups (*p* < 0.05; Figure 5A,G). Masson staining images and quantitative analysis revealed that the WD + rMfp6-1 group formed significantly larger granulation tissue areas than the control and WD groups (*p* < 0.05; Figure 5B,H). Further observation of collagen deposition revealed denser and more collagen content in the WD + rMfp6-1 group than in the control and WD groups (Figure 5C–E). Quantitative analysis of collagen content via image analysis confirmed that the collagen deposition ratio in the WD + rMfp6-1 group (82.07%) was significantly higher than that in the control group (25.93%) and the WD group (51.97%) (*p* < 0.05; Figure 5I). The number of newly formed blood vessels in the WD + rMfp6-1 group was significantly higher than in the control group and the WD group (*p* < 0.05; Figure 5J).

### 2.5. Immunohistochemical Analysis Reveals That WD + rMfp6-1 Inhibits Inflammation and Promotes Angiogenesis

**Figure 6:**
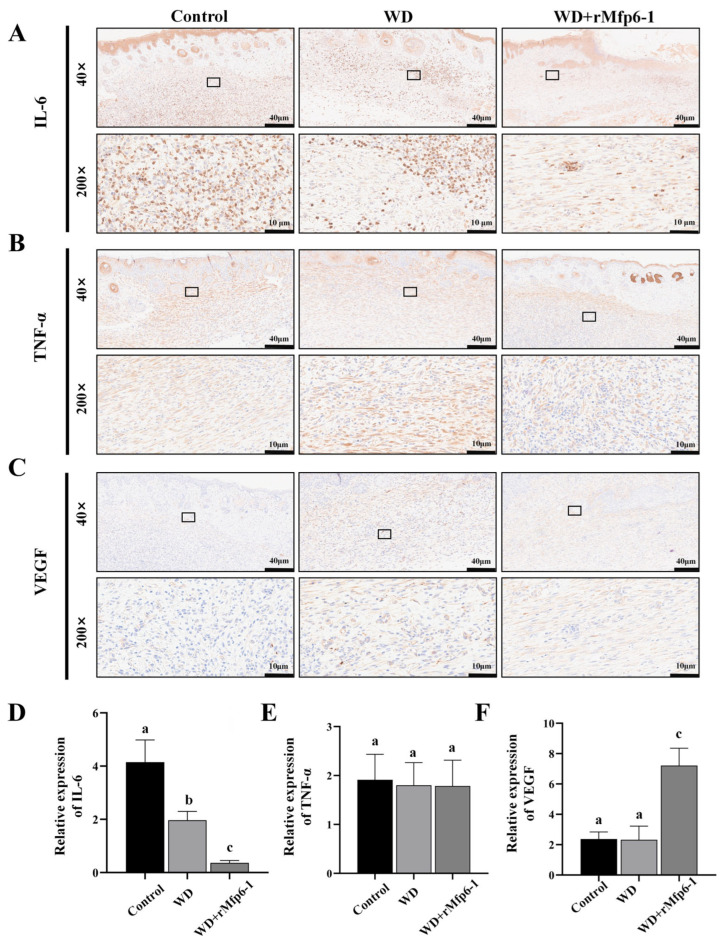
Effects of rMfp6-1 on inflammatory modulation and vascularization within wound sites. (**A**–**C**) Immunohistochemical staining of IL-6 (**A**), TNF-α (**B**), and VEGF (**C**) in wound tissues from the Control, WD, and WD + rMfp6-1 groups. Upper panels show panoramic views (40×), and lower panels show higher-magnification local views (200×). Positive immunoreactivity is indicated by brown staining. (**D**–**F**) Quantitative analysis of the positive staining area for IL-6 (**D**), TNFα (**E**), and VEGF (**F**), derived from 48 stochastic microscopic fields. Results are expressed as mean ± SD. Distinct alphabetical superscripts over the bars signify substantial differences at a threshold of *p* < 0.05. Results are expressed as mean ± SD (*n* = 6).

Immunohistochemical analysis was performed to elucidate the molecular basis of rMfp6-1-mediated wound repair, focusing on inflammation and angiogenesis (Figure 6). Specifically, we examined the expression profiles of IL-6, TNF-α, and VEGF within granulation tissues. Quantitative assessment demonstrated that rMfp6-1 treatment significantly suppressed IL-6 levels compared to both the control and WD groups (*p* < 0.05; Figure 6A,D). No significant difference in TNF-α staining intensity was observed among the groups (*p* > 0.05; Figure 6B,E). Conversely, VEGF expression was markedly upregulated in the WD + rMfp6-1 group relative to other cohorts (*p* < 0.05; Figure 6C,F), suggesting a pro-angiogenic mechanism.

## 3. Discussion

In this study, we successfully identified, cloned, and expressed a mussel foot protein, Mfp6-1, from the mussel *M. coruscus*, and elucidated its potential application as a bioactive wound dressing. While mussel foot proteins have traditionally been studied in the context of underwater adhesion, our results suggest that rMfp6-1 exhibits bioactivities of potential relevance to wound repair in a murine model. The rMfp6-1 exhibited potent antioxidant activity dependent on its cysteine residues and, when applied in vivo, significantly accelerated wound healing. These findings suggested that the biotechnological potential of Mfp6-1, a mussel byssal protein with antioxidant properties from *M. coruscus*, as a mussel-derived bioactive protein for skin wound repair. In the murine full-thickness skin wound model, rMfp6-1 treatment promoted wound closure and tissue regeneration and was accompanied by enhanced angiogenesis-related responses.

Sequence analysis of *M. coruscus* Mfp6-1 revealed unique structural features distinguishing it from other well-characterized mussel adhesive proteins (MAPs), such as Mfp-3 and Mfp-5, which are typically defined by high DOPA content and intrinsic disorder [18]. In contrast, Mfp6-1 is characterized by relatively high cysteine (11.0%) and tyrosine (~16.5%) contents, and structural prediction suggests a compact cysteine-rich architecture containing short β-structural elements connected by turns and coil regions. This specific protein structure holds significant functional implications, with the abundance of cysteine being particularly noteworthy. In the context of mussel biology, cysteine-rich proteins like Mfp-6 are hypothesized to maintain the redox balance within the byssal plaque, potentially reducing oxidized DOPA-quinone back to DOPA to preserve adhesion [9]. Based on the AlphaFold predicted model, Cys60, Cys89, and Cys95 were potentially exposed cysteine residues (Figure 1D). However, considering the spatial proximity between Cys60 and Cys89 and the inherent uncertainty of the predicted model, they may also constitute an additional disulfide-bonding pair. In our recombinant system, although rMfp6-1 possesses a well-defined and tightly packed structure, its observed antioxidant activity is likely attributed to a specific subset of cysteine residues that remain solvent-accessible as free thiol groups. This mechanism is further substantiated by our experimental evidence from both alkylation (IAM) and free thiol (DTNB) assays, which confirm the presence and functional necessity of these available functional groups within the compact fold. Nevertheless, these observations mainly reflect the behavior of recombinant rMfp6-1 under the present in vitro conditions, while the folding state of native Mfp6-1 remains to be further clarified.

Our in vitro experiments confirmed the robust antioxidant capacity of rMfp6-1. Alkylation assays provided direct mechanistic evidence: blocking thiol groups significantly attenuated DPPH radical scavenging activity, confirming cysteine residues as the primary driver of this function. Consistent with this interpretation, DTNB-based thiol quantification showed that rMfp6-1 contained accessible free sulfhydryl groups, whereas IAM treatment markedly reduced the DTNB-detectable signal. Intriguingly, the DTNB-quantified five free thiols slightly exceeded the three sites predicted by AlphaFold. This discrepancy may reflect differences between the predicted structural model and the folding state of recombinant rMfp6-1 under the present in vitro conditions. However, cysteine abundance alone may not fully account for its potency. Notably, a recently identified MFP-20 from *M. coruscus* contains a higher cysteine content (16%) than Mfp6-1 (11%), yet the DPPH assay reported antioxidant efficacy at the concentration of 3.2 mM of MFP-20 (<80%) [15] was lower than Mfp6-1 (>90%; present study). This suggests a more complex underlying mechanism. While cysteine is the primary ROS scavenger, Mfp6-1 also retains an exceptionally high content of tyrosine residues. Tyrosine itself is redox-active and can participate in electron transfer processes [19]. Given the difference in tyrosine content between MFP-20 and Mfp6-1, we hypothesize that Mfp6-1 exploits a specific Cys-Tyr synergy lacking in the former. One possible explanation is that the abundant tyrosine residues in Mfp6-1 participate in a relay network that helps maintain the redox state of cysteine residues through long-range electron transfer [20]. It has been proposed that the side chains of tyrosine and cysteine can function as ‘relay stones’ for electron transfer—a process driven by the deprotonation of the tyrosyl ring oxygen and cysteinyl sulfur, which lowers their reduction potentials [21]—which may render Mfp6-1 more readily oxidizable than MFP-20. Nevertheless, this hypothesized cooperative mechanism warrants further in-depth investigation.

In vivo evaluation demonstrated that the rMfp6-1-loaded dressing (WD + rMfp6-1) significantly accelerated wound closure compared to the control group. The dressing vehicle (WD) itself exhibited improved healing kinetics relative to the control, likely due to its physicochemical properties, which provide hydration and prevent desiccation [22,23]. However, the decisive therapeutic advantage of rMfp6-1 over the vehicle alone was primarily manifested in the quality of tissue regeneration. Histological analysis revealed that, compared to the WD group, rMfp6-1 treatment resulted in enhanced re-epithelialization, thicker granulation tissue, and, crucially, denser and more hierarchically organized collagen deposition. Effective wound repair requires not only rapid closure but also the restoration of extracellular matrix (ECM) integrity, a process heavily reliant on fibroblast activity [24]. While the WD provided a foundational physical environment for closure, the incorporation of rMfp6-1 biologically augmented the repair effects.

Wound healing is a complex process involving distinct but overlapping phases: hemostasis, inflammation, proliferation, and remodeling [25]. Prolonged inflammation is often detrimental to healing. To further characterize the response of the murine wound model to rMfp6-1 treatment, we examined representative markers (IL-6, TNF-α, and VEGF) related to inflammation and angiogenesis. Immunohistochemical analysis revealed that rMfp6-1 treatment significantly downregulated IL-6 expression compared to the control (Figure 6), suggesting a potent anti-inflammatory effect that may accelerate the transition from the inflammatory phase to proliferation. Notably, while IL-6 levels were markedly reduced, TNF-α expression remained comparable between the groups, indicating that rMfp6-1 might selectively modulate specific inflammatory signaling pathways. This is likely a downstream effect of rMfp6-1’s antioxidant activity, as ROS scavenging prevents the activation of oxidative stress-induced inflammatory pathways (such as NF-κB) [26,27]. Furthermore, we observed a significant upregulation of VEGF, a pivotal factor for angiogenesis. Consistent with this molecular finding, histological examination of the granulation tissue revealed a markedly higher density of neovascularization in the rMfp6-1-treated group compared to controls. This robust angiogenic response is critical, as the newly formed capillary network provides the oxygen and nutrients necessary to sustain the high metabolic demands of proliferating fibroblasts and collagen synthesis [28,29]. It is plausible that by reducing oxidative stress and inflammation, rMfp6-1 creates a permissive environment that facilitates the transition from the inflammatory phase to the proliferative phase, thereby enhancing angiogenesis and collagen synthesis. Although we have demonstrated in vitro that *M. coruscus* rMfp6-1 possesses antioxidant activity and detectable free sulfhydryl groups, the precise cellular and molecular mechanisms underlying its potential biomedical wound-healing effects in mammalian models remain to be further investigated.

In summary, this study characterized a cysteine-rich mussel foot protein and evaluated the bioactivity of rMfp6-1 in a murine wound-healing model. Attributed primarily to the reducing environment provided by its cysteine residues, rMfp6-1 supported the healing process, as evidenced by improved vascularization and collagen deposition. These findings support further exploration of rMfp6-1 as a candidate bioactive component for wound-healing formulations, with potential applications in higher mammals.

## 4. Materials and Methods

### 4.1. Sequence Analysis of Mfp6-1 from M. coruscus

The coding sequence of Mfp6-1 (GenBank: KP876477.1) was retrieved from the *M*. *coruscus* transcriptome. The open reading frame was identified using (Open Reading Frame) ORF Finder, the signal peptide was predicted using SignalP-6.0 (https://services.healthtech.dtu.dk/services/SignalP-6.0/, accessed on 25 September 2025), and the theoretical molecular weight and isoelectric point (pI) of the mature peptide were calculated using ProtParam (http://web.expasy.org/protparam/, accessed on 25 September 2025). Homologous proteins were identified by Blastp against the NCBI non-redundant protein database, and representative *Mytilus* homologs were selected for comparison (http://blast.ncbi.nlm.nih.gov/Blast.cgi, accessed on 6 April 2026). Multiple sequence alignment of Mfp6-1 homologs was performed using the MUSCLE algorithm (https://www.ebi.ac.uk/Tools/msa/muscle/, accessed on 24 April 2026) and subsequently visualized and annotated using Jalview (v2.10; https://www.jalview.org/, accessed on 6 April 2026). Conserved domains and sequence features were analyzed using the SMART web server (http://smart.embl-heidelberg.de, accessed on 6 April 2026).

All subsequent analyses were performed using the mature peptide sequence after removal of the signal peptide. The amino acid composition of mature Mfp6-1 was calculated from the signal peptide-cleaved sequence. The mature peptide sequence was submitted to AlphaFold for three-dimensional structure prediction [30]. Model confidence was evaluated using pLDDT. The spatial distribution of cysteine residues in the predicted model was further inspected in PyMOL 3.1.8.

### 4.2. Recombinant Expression, Purification, and Refolding of rMfp6-1

To ensure efficient expression in *E. coli*, the mature Mfp6-1 sequence was codon-optimized and synthesized via GenScript’s OptimumGene™ platform (https://www.genscript.com/tools/gensmart-codon-optimization, accessed on 25 September 2025). This fragment was then inserted between the *Nco I* and *Xho I* sites of the pET-28a (+) vector, facilitating the expression of an N-terminal 6 × His-tagged fusion protein.

Recombinant protein production was induced by adding 1 mM IPTG when the culture density (OD600) reached 0.6–0.8, followed by incubation under two conditions: 30 °C for 6 h and 25 °C for 10 h. The target recombinant protein was mainly recovered in the inclusion-body fraction after cell disruption. After harvesting the biomass through centrifugation (1000× *g*, 15 min), the cells were resuspended in PBS and disrupted using ultrasonic treatment. The resulting inclusion bodies were isolated (8000× *g*, 10 min) and dissolved in a urea-based binding buffer (8 M urea, 20 mM Tris-HCl, 10 mM imidazole, pH 8.0). Purification was performed using Ni-NTA affinity chromatography under denaturing conditions, with the protein being recovered using an elution buffer containing 300 mM imidazole.

The denatured protein (0.5 mg/mL) underwent a gradient dialysis process at 4 °C to restore its native conformation. This involved stepwise reduction of urea concentration (from 6 M to 0 M) in a refolding environment (20 mM Tris-HCl, pH 8.0) supplemented with GSH/GSSG (0.9 mM/0.1 mM). The final product was dialyzed against PBS, lyophilized, and preserved at −20 °C. The purity and molecular identity were verified by 12% SDS-PAGE and Western blotting, respectively. The expression yield was estimated by comparison with bovine serum albumin (BSA) standards on SDS-PAGE, and the final recovery after purification and refolding was calculated from the lyophilized protein mass obtained after the final dialysis step relative to the initial culture volume. Anti-His-tag primary antibodies and HRP-conjugated secondary antibodies (Abclonal, Wuhan, China) were used for western blot analysis, with signals visualized through ECL detection.

### 4.3. Mass Spectrometry Analysis

The lyophilised rMfp6-1 was reconstituted in an 8 M urea solution containing protease inhibitors, with its concentration quantified using a BCA protein assay kit (Beyotime, Nanjing, China). To prepare the sample for MS, 50 μg of the protein was reduced with 10 mM dithiothreitol (DTT) (56 °C, 30 min) and subsequently alkylated with 20 mM iodoacetamide in the dark for 30 min. After lowering the urea molarity to <2 M with 100 mM NH_4_HCO_3_, trypsin was introduced at a 1:50 (*w*/*w*) ratio for an overnight incubation at 37 °C. The digested peptides were then purified using C18 StageTips and resuspended in 0.1% formic acid after vacuum drying.

Peptide separation was performed on a nano-flow C18 column coupled to a Q Exactive HF-X mass spectrometer (Thermo Fisher Scientific, Waltham, MA, USA) via a nano-ESI source. We applied a 120-min linear gradient of 0.1% formic acid in acetonitrile at 300 nL/min for optimal separation. Mass spectra were collected in data-dependent acquisition (DDA) mode, featuring a primary MS scan (350–1500 *m*/*z*, 60,000 resolution) followed by HCD-based MS/MS of the 20 most abundant precursors. Raw files were analyzed through the MaxQuant platform (v1.6.17.0), searching against the UniProt database (http://www.uniprot.org, accessed on 19 October 2025). During the search, carbamidomethylation of cysteine was defined as a fixed modification, while methionine oxidation was considered variable. A threshold of ≤1% false discovery rate (FDR) was maintained for both protein and peptide identification.

### 4.4. Antioxidant Activity Assay

To evaluate the antioxidant potential, a DPPH radical-scavenging kit (Solarbio, Beijing, China) was utilized following the established protocol [31]. Three experimental groups—rMfp6-1, iodoacetamide-alkylated rMfp6-1 (rMfp6-1-IAM), and L-ascorbic acid (serving as a positive control)—were separately reacted with a 0.02% DPPH solution prepared in anhydrous ethanol. After the reaction, the absorbance at 517 nm was monitored with a Synergy H1 spectrophotometer (BioTek, Winooski, VT, USA). The radical-scavenging efficiency was subsequently determined based on the following equation:

$$\mathrm{Scavenging}\mathrm{Activity}\;(\%)=\frac{A_{0}-A_{2}+A_{1}}{A_{0}}\times 100$$

In the aforementioned calculation, the parameters are defined as follows: ***A*0** represents the background absorbance (DPPH in anhydrous ethanol and water), ***A*1** denotes the control absorbance (protein sample in ethanol without DPPH), and ***A*2** is the absorbance of the test group (protein sample combined with DPPH). All statistical evaluations and figure generations were performed using GraphPad Prism 9.5.0.

The alkylated derivative was synthesized by reacting rMfp6-1 with 20 mM iodoacetamide within a 50 mM NH_4_HCO_3_ environment in the dark. To eliminate unreacted materials, the mixture underwent extensive dialysis against ultrapure water for 24 h using a 1 kDa molecular weight cutoff membrane.

### 4.5. Determination of Accessible Free Sulfhydryl Groups in rMfp6-1

Accessible free sulfhydryl groups in rMfp6-1 were determined using a Free Sulfhydryl Assay Kit (DTNB method; Beyotime, Nanjing, China) [32]. Briefly, the DTNB stock solution and Ellman’s reagent solution were prepared as instructed by the kit. In a 96-well plate, 180 μL of Ellman’s reagent solution was added to each well, followed by 20 μL of protein sample or standard. Each group was measured in triplicate. After incubation at room temperature (20–25 °C) for 15 min, absorbance was measured at 412 nm using a microplate reader. Blank-corrected absorbance values were calculated using the blank control, and sulfhydryl concentration was determined from the GSH standard curve.

For concentration-dependent analysis, rMfp6-1 samples were adjusted to final concentrations of 1, 2, 3, and 5 mg/mL before DTNB measurement. For alkylation treatment, rMfp6-1 samples were pre-incubated with 20 mM iodoacetamide (IAM) in the dark for 1 h and then subjected to the same DTNB assay. Results were expressed as μmol/mL. This assay was employed to selectively quantify surface-accessible or sterically exposed free sulfhydryl groups, as opposed to the total cysteine content within the protein.

### 4.6. Preparation of Liquid Wound Dressing

The preparation method of the liquid wound dressing is as follows: Glycerol (15%, *w*/*w*) and propylene glycol (10%, *w*/*w*) are first dissolved in deionized water (approximately 58%, *w*/*w*), and gently stirred. A gel matrix was then formed by sequentially adding methylcellulose (10%, *w*/*w*), sodium alginate (2%, *w*/*w*), and gum arabic (1%, *w*/*w*), ensuring that each component was fully dissolved before adding a polyvinyl alcohol (PVA, 2%, *w*/*w*) solution. Finally, rMfp6-1 was incorporated into the dressing after pH adjustment and cooling. A total of 10 g dressing was prepared, and rMfp6-1 was added at a final concentration of 1% (*w*/*w*), corresponding to 100 mg protein in 10 g dressing. In this system, the protein concentration was 10 mg/mL, equivalent to 0.83 mM based on a molecular weight of 12.04 kDa. The dressing was left to stand at room temperature for 20–30 min and then stored at 4 °C.

### 4.7. Animal Experiment and Wound-Healing Evaluation

Male BALB/c mice, aged five weeks and weighing approximately 20 ± 2 g, were obtained from SPF Biotechnology Co., Ltd (Beijing, China). The housing environment was strictly regulated at a constant temperature of 22 ± 2 °C and a relative humidity of 50–60%, following a 12 h light/dark circadian rhythm. Prior to any experimental procedures, the mice were provided with a one-week stabilization period to adapt to their surroundings, during which they had unrestricted access to standard chow and water. All experimental protocols were designed in compliance with the Guidelines for the Care and Use of Laboratory Animals and received ethical clearance from the Animal Ethics Committee of Shanghai Ocean University (No. SHOU-DW-2022-016).

Mice were anesthetized by intraperitoneal injection of tribromoethanol (Avertin; 1.25% *w*/*v*, 200 μL/10 g body weight). The dorsal hair was shaved, and the skin was disinfected with 75% ethanol. A circular, 1-cm-diameter full-thickness excisional wound was created on the dorsal midline using a sterile scalpel, ensuring complete removal of the panniculus carnosus.

Mice were randomly allocated into three experimental cohorts (n = 6 per group): a control group (C), which received no intervention; a vehicle group (WD), treated with the wound dressing alone; and an rMfp6-1 treatment group (WD + rMfp6-1), treated with the rMfp6-1-containing dressing. In the dressing, rMfp6-1 was present at a final concentration of 1% (*w*/*w*), equivalent to 10 mg/mL (0.83 mM). A total of 500 μL of dressing was applied to each mouse, corresponding to a total dose of 5 mg rMfp6-1 per application for the WD + rMfp6-1 group. These topical applications were administered daily for 7 consecutive days. To monitor the healing progress, digital photographs of the wounds were taken on days 0, 3, 5, and 7. To minimize bias caused by minor differences in initial wound geometry, the actual wound margin of each mouse was manually outlined and the wound area was measured using ImageJ software 1.37 (Bethesda, MD, USA) at day 0 and each subsequent time point (days 3, 5, and 7). Wound closure percentage was then calculated by comparing the wound area at each time point (At) with the actual wound area of the same mouse at day 0 (A0), according to the following formula [33]:

$$\mathrm{Wound}\mathrm{closure}\;(\%)=\frac{A_{0}-A_{t}}{A_{0}}\times 100$$

where A0 is the actual wound area of the same mouse on day 0, and At is the wound area of that mouse at days 3, 5, and 7. On day 7, all animals were euthanized, and wound tissues were harvested for further analyses.

### 4.8. Histological Analysis

Wound tissues were fixed in 4% paraformaldehyde for 24 h, dehydrated through a graded ethanol series, cleared in xylene, and embedded in paraffin. Serial sections (5 μm) were cut using a Leica RM2235 microtome (Wetzlar, Germany). Sections were stained with hematoxylin and eosin (H&E) to assess re-epithelialization, and with Masson’s trichrome to evaluate collagen deposition. Images were captured using a Nikon Eclipse Si light microscope (Tokyo, Japan). Quantitative analysis of newly formed epidermis, granulation tissue area, and collagen content was performed in eight randomly selected fields per sample using ImageJ software. For quantification of collagen deposition, six mice were included in each group. One section was prepared per mouse, and eight random microscopic fields within the granulation tissue region were analyzed per section, resulting in a total of 48 fields per group.

### 4.9. Immunohistochemical (IHC) Analysis

Tissue sections (5 μm thick) were deparaffinized and rehydrated through a graded alcohol series. Heat-induced epitope retrieval was performed in citrate buffer (pH 6.0) at 95 °C for 20 min, followed by the inactivation of endogenous peroxidase using 3% H_2_O_2_ for 10 min. The slides were incubated overnight at 4 °C with primary antibodies targeting IL-6 (1:200), TNF-α (1:500), and VEGF (1:100) (all from Abclonal, Wuhan, China). Detection was facilitated by an HRP-conjugated secondary antibody (1:200, Servicebio, Wuhan, China) for 30 min at ambient temperature. Immunoreactivity was visualized using a DAB chromogen kit, followed by hematoxylin counterstaining for nuclear visualization. The sections were examined under a Nikon light microscope, and the positive-stained regions were quantified as a percentage of the total area using ImageJ. For each immunohistochemical marker, six mice were included in each group. One section was prepared per mouse, and eight random microscopic fields within the granulation tissue region were analyzed per section, resulting in a total of 48 fields per group.

### 4.10. Statistical Analysis

Data are expressed as mean ± SD and analyzed using GraphPad Prism 9.5.0. Normality and homogeneity of variance were assessed using the Shapiro-Wilk test. For data meeting these assumptions, an unpaired one-way ANOVA with Tukey’s post-hoc test was used. Alternatively, when data did not meet the requirements of normality and homogeneity, the Wilcoxon test was used for analysis. Statistical significance was defined as *p* < 0.05.

## Data Availability

Data will be made available upon request.
